# Supplementary material for: A high-resolution mRNA expression time course of embryonic development in zebrafish
Source: eLife. 2017 Nov 16;6:e30860. doi: 10.7554/eLife.30860 (PMC5690287; doi:10.7554/eLife.30860)
Supplement: Supplementary file 6. [file elife-30860-supp6.zip › biolayout-clusters-files/Cluster083-genes.html]

Cluster083


# Cluster083: Genes

| Ensembl ID | Gene Name | Chr | Start | End | Biotype |
| --- | --- | --- | --- | --- | --- |
| ENSDARG00000061850 | CR847986.1 | 7 | 20223820 | 20250264 | protein\_coding |
| ENSDARG00000068263 | csf1b | 8 | 37136136 | 37169983 | protein\_coding |
| ENSDARG00000015076 | cx44.1 | 1 | 46754800 | 46757618 | protein\_coding |
| ENSDARG00000089086 | mustn1 | 11 | 18020167 | 18023521 | protein\_coding |
| ENSDARG00000067997 | myhz1.3 | 5 | 31662346 | 31674230 | protein\_coding |
| ENSDARG00000015876 | npas3b | 15 | 8448640 | 8541484 | protein\_coding |
| ENSDARG00000076411 | ptrfa | 3 | 16826295 | 16862540 | protein\_coding |
| ENSDARG00000055518 | pygma | 21 | 28408357 | 28435544 | protein\_coding |
| ENSDARG00000016088 | rtn2a | 15 | 9034820 | 9055925 | protein\_coding |
| ENSDARG00000078598 | si:ch1073-184j22.2 | 2 | 5485182 | 5492830 | protein\_coding |
| ENSDARG00000070320 | si:dkey-245p14.4 | 1 | 18842207 | 18844422 | protein\_coding |
| ENSDARG00000093940 | si:dkeyp-77c8.1 | 13 | 37046086 | 37054925 | protein\_coding |
| ENSDARG00000063288 | slc2a11b | 5 | 9509966 | 9541545 | protein\_coding |
| ENSDARG00000103789 | tmem130 | 12 | 17744228 | 17752733 | protein\_coding |
| ENSDARG00000062341 | wfs1a | 1 | 13627138 | 13653114 | protein\_coding |
